# Predictors of Response to Oral Medications and Low-Histamine Diet in Patients with Chronic Urticaria

**DOI:** 10.1155/2022/5243825

**Published:** 2022-02-22

**Authors:** Hui-Ling Chiang, Chen-Hung Chen, Malcolm Koo, Tzung-Yi Tsai, Cheng-Han Wu

**Affiliations:** ^1^Division of Immunology, Allergy and Rheumatology, Dalin Tzu Chi Hospital, Buddhist Tzu Chi Medical Foundation, Dalin, Chiayi, Taiwan; ^2^Division of Allergy, Immunology and Rheumatology, Taipei Tzu Chi Hospital, Buddhist Tzu Chi Medical Foundation, School of Medicine, Tzu Chi University, Hualien, Taiwan; ^3^Graduate Institute of Long-term Care, Tzu Chi University of Science and Technology, Hualien City, Hualien, Taiwan; ^4^Department of Medical Research, Dalin Tzu Chi Hospital, Buddhist Tzu Chi Medical Foundation, Dalin, Chiayi, Taiwan; ^5^Department of Environmental and Occupational Health, College of Medicine, National Cheng Kung University, Tainan City, Taiwan; ^6^Department of Nursing, Tzu Chi University of Science and Technology, Hualien City, Hualien, Taiwan

## Abstract

**Background:**

Chronic urticaria (CU) is comprised of diverse phenotypes, and thus, a shift towards a precision medical approach is warranted in its management.

**Methods:**

This study enrolled 78 patients with CU. Serum erythrocyte sedimentation rate, hemoglobin, hematocrit, eosinophil count, IgE, antinuclear antibody (ANA), and serum diamine oxidase (DAO) levels of the patients were measured and were compared according to the patient's response to second-generation antihistamines (sgAH), corticosteroids, leukotriene receptor antagonist (LTRA), H_2_ blockers, and low-histamine diet.

**Results:**

Age- and sex-adjusted logistic regression analysis showed that patients with duration of CU > 3 years (adjusted odd ratio [aOR] = 4.39) and a DAO level < 10 U/mL (aOR = 3.90) were significantly associated with a good sgAH response. Age > 50 years (aOR = 0.02), duration of chronic urticaria > 3 years (aOR =0.06), and an ANA titer ≥ 1 : 80 (aOR = 0.03) were significantly and inversely associated with corticosteroid response. A low-histamine diet response was significantly associated with LTRA response (aOR = 67.29). In addition, a DAO level < 5.4 U/mL (aOR = 71.95) was significantly associated with H_2_ blocker response. Furthermore, concomitant angioedema (aOR = 10.56), multiple food triggers (aOR = 11.69), and a DAO level < 5.4 U/mL (aOR = 3.78) were significantly associated with a low-histamine diet response. Conversely, dermatographic urticaria and a hematocrit level < 36% were significantly and inversely associated with low-histamine diet response.

**Conclusions:**

Several promising biomarkers were identified in this study to predict the efficacy of chronic urticaria treatment. DAO could be a novel biomarker for predicting the efficacy not only of dietary intervention but also for antagonists of H_1_ and H_2_ receptors.

## 1. Introduction

Chronic urticaria (CU) has traditionally been defined as episodes of urticaria rash that occur almost daily for more than six weeks. CU is a common disease and is prevalent in approximately 1% of the general population. However, treatment options are limited. The recommended treatment outlined in the CU guidelines includes second-generation H1 antihistamines (sgAH), omalizumab, and cyclosporine [1, 2]. Due to the cost of omalizumab and the adverse effects of cyclosporin, most patients with CU choose sgAH as their only treatment. However, many of them are dissatisfied with sgAH monotherapy because it cannot eliminate all symptoms. Furthermore, a number of patients are unable to tolerate sgAH because of its sedating effects, even at low doses. Additionally, CU is comprised of different phenotypes. For example, 40% of patients with CU have concomitant angioedema, 20% have CU that is physically triggered, and some have long-lasting wheals. Standard care may not always fit all phenotypes. Thus, there is a need to shift toward a precision medicine approach in management of CU.

Diamine oxidase (DAO) is the main enzyme involved in histamine catabolism, and DAO deficiency has been suspected as a probable cause of histamine intolerance. Beyond a critical level, histamine can induce a variety of symptoms in individuals with histamine intolerance, including urticaria, migraine, and gastrointestinal upset. A significant inverse correlation (*r* = −0.335, *p* = 0.001) between DAO and serum histamine level was reported by Cho et al. [3]. However, the application of serum DAO levels in CU management has yet to be determined. Therefore, the aim of this study was to investigate the predictors of treatment efficacy for oral medications and dietary interventions for patients with CU. The role of serum DAO levels in CU management was also explored.

## 2. Materials and Methods

### 2.1. Characteristics of the Patients and Treatment

Adult patients 18 years of age or older with CU were recruited from two regional hospitals in Taiwan. The diagnosis of CU was based on the definition given in current CU guideline [1, 2]. All participants were asked about the duration of their CU and whether they had concomitant angioedema, the duration of wheals, and exacerbation of CU with certain foods. All patients were prescribed up to a twofold dose of sgAH and were asked to follow the recommendations of a low-histamine diet. Montelukast and H_2_ blocker was also prescribed if necessary. Low-dose corticosteroids were used only to relieve symptom. Patients who can achieve symptom free by regular antihistamine treatment were defined as good response to sgAH; those who reported improvement but not symptom free were defined as partial response to sgAH; and those who reported no improvement were defined as no response. The response to montelukast, H_2_ blocker, or a low-histamine diet was evaluated by a decrease in sgAH for individuals with good response to sgAH. All participants signed informed consent according to a study protocol approved by the Institutional Review Board of Dalin Tzu Chi Hospital, Buddhist Tzu Chi Medical Foundation (No. B10902014-2) or Taipei Tzu Chi Hospital, Buddhist Tzu Chi Medical Foundation (No. 09-C-095).

### 2.2. Measurement of Serum DAO and Laboratory Parameters

The following laboratory tests were performed as follows: serum erythrocyte sedimentation rate (ESR), hemoglobin (Hb) and hematocrit (Hct) levels, eosinophil count, IgE, and antinuclear antibody (ANA) and DAO levels. The serum was separated and kept at −20°C until needed. Laboratory blood parameters and serum DAO levels were measured in the following way. Briefly, total and specific IgE were analyzed by fluorimetric enzyme-linked assay (FEIA) (Phadia™, Thermo Fisher, Uppsala, Sweden). DAO levels were measured using the DAO ELISA kit (Immundiagnostik AG, Bensheim, Germany), according to the manufacturer's protocol. A dose-response curve of optical density versus a standard concentration was generated, using the values obtained from the standard. The reference values from the manufacturer's specification for DAO are as follows: histamine intolerance is highly likely in patients with DAO activity < 3 U/mL, likely in patients with DAO activity < 10 U/mL, and improbable in patients with DAO activity > 10 U/mL. Other index data were obtained from the routine laboratory examinations.

### 2.3. Statistical Analysis

Comparative analyses were conducted using chi-square tests for categorical variables and independent *t*-test for continuous variables. Pearson's correlations were performed to analyze the correlation between continuous variables. To identify significant predictors of treatment efficacy in patients with CU, five separate age- and sex-adjusted logistic regression analyses were performed with the following dependent variables: good response to sgAH, corticosteroid response, leukotriene receptor antagonist response, H_2_ blockers, and low-histamine diet response. When the logistic regression model failed to converge for binary outcomes, firth logistic regression was used [4]. The procedure was implemented through the IBM SPSS extension command STATS FIRTHLOG. A *p* value < 0.05 was considered statistically significant. All statistical analyses were conducted using IBM SPSS version 28.0 (IBM Corp., Armonk, NY, USA).

## 3. Results

The basic characteristics of the patients with CU are shown in Table 1. A total of 78 patients with CU completed the study. Of them, 54 were females (69.2%) and the overall mean age was 45.8 years (range: 19 to 74 years). The mean level of DAO was 8.91 U/mL, and the range was 1.14 U/mL to 63.15 U/mL. In addition, Table 2 shows that DAO levels were significantly lower in male patients than in their female counterparts (*p* = 0.004). They were significantly lower in patients with CU who were >50 years of age compared with those ≤ 50 years of age (*p* = 0.033). Patients with dermatographic urticaria had a significantly lower DAO compared to those with chronic spontaneous urticaria (*p* = 0.006). Moreover, the mean ESR level was significantly lower in patients with dermatographic urticaria compared to those with chronic spontaneous urticaria (mean 5.36 and SD 3.73 mm/hr vs mean 11.17 and SD 9.58 mm/hr, *p* = 0.001, respectively). DAO was also significantly correlated to ESR (*p* = 0.004) and inversely correlated with Hb (*p* = 0.022) and Hct (*p* = 0.016).

Of the 76 patients with available sgAH treatment response, 44 (57.9%) reported having a good response, 30 reported having a partial response (39.5%), and 2 reported no response (2.6%). The results of the multiple logistic regression analysis showed that a significantly favorable response to sgAH was observed in patients who had CU > 3 years (adjusted odds ratio [aOR] = 4.39, 95% confidence interval [CI] 1.14–16.99, *p* = 0.032) and a DAO level < 10 U/mL (aOR = 3.90, 95% CI 1.07–14.25, *p* = 0.039).

Of the 20 patients who needed oral corticosteroid treatment for symptomatic relief, 14 (70%) reported a good response, and 6 (30%) reported a poor response. The results of the multiple logistic regression analysis showed that a significantly reduced risk of corticosteroid response was observed in patients with CU who had a ANA titer ≥ 1 : 80 (aOR = 0.03, 95% CI 0.00–0.39, *p* = 0.005), > 50 years of age (aOR = 0.02, 95% CI 0.01–0.92, *p* = 0.041), and duration of CU > 3 years (aOR = 0.06, 95% CI 0.00–0.96, *p* = 0.047).

Of 11 patients with available montelukast response, six reported having a good response (54.5%), and 5 reported having a poor response (45.5%). The results of the multiple logistic regression analysis showed that a significantly higher risk of montelukast response was observed in patients with CU who benefited from a low-histamine diet (aOR = 67.29, 95% CI 2.07–64667, *p* = 0.012).

Of eight patients with available H_2_ blocker response, three (37.5%) reported having a good response, and 5 (62.5%) reported a poor response. The results of the multiple logistic regression analysis showed that patients with CU who had a DAO level < 5.4 U/mL (the median of DAO levels) were significantly more likely to respond to H_2_ blockers (aOR = 71.95, 95% CI 1.55–89411, *p* = 0.027).

Among the 72 individuals who had followed a low-histamine diet recommendation, 55 (70.5%) reported improvement of CU symptoms. The results of the multiple logistic regression analysis showed that a significantly higher risk of low-histamine diet response was observed among patients with concomitant angioedema (aOR = 10.56, 95% CI 1.28–1374.71, *p* = 0.024). In addition, patients with multiple food triggers were more likely to benefit from a low-histamine diet (aOR = 11.69, 95% CI 1.42–96.35, *p* = 0.022). Moreover, patients with CU who had a DAO level < 5.4 U/mL, which was the median of DAO levels, were significantly more likely to respond to a low-histamine diet (aOR = 3.78, 95% CI 1.06–13.46, *p* = 0.040). Conversely, patients with dermatographic urticaria were less likely to significantly benefit from a low-histamine diet (aOR = 0.23, 95% CI 0.06–0.86, *p* = 0.029). Patients with CU who had an Hct level < 36% were less likely to benefit from a low-histamine diet (aOR = 0.19, 95% CI 0.04–0.80, *p* = 0.024) (Table 3).

Among the 78 participants, the most common food trigger reported was shrimp (*n* = 12), followed by alcohol (*n* = 6), peanuts (*n* = 5), and processed food with food additives (*n* = 5). These foods were included in this study due to intermittent eruptions despite the elimination of culprit foods. Four individuals with a shrimp trigger had specific IgE (sIgE) to shrimp detected. No individuals with the peanut trigger had sIgE to peanut detected. Patients with exacerbations after consuming shrimp were more likely to benefit from a low-histamine diet (aOR = 11.28, 95% CI 1.29–1489.21, *p* = 0.024). Patients with exacerbation after pumpkin consumption were less likely to respond to a low-histamine diet (aOR = 0.06, 95% CI 0.00–0.79, *p* = 0.032). In the subgroup with DAO levels below 5.4 U/mL, patients who had reactions to processed foods with food additives had significantly lower DAO levels (mean 1.80 and SD 0.88 U/mL vs mean 3.58 and SD 1.11 U/mL, *p* = 0.004). The prevalence of angioedema was significantly higher in patients who had exacerbation of symptoms after consuming peanuts (3/5; 60% vs 10/73; 13.70%; *p* = 0.030). Patients with exacerbations after consuming plant-derived foods were more likely to have contact dermatitis with culprit foods or herbal medicines (4/24; 16.67% vs 1/54; 1.85%; *p* = 0.029). The prevalence of eczema was higher among patients who had exacerbations with alcohol consumption (2/6; 33.33% vs 5/72; 6.94%; *p* = 0.030). Patients with eruptions triggered predominantly by heat were more likely to have exacerbations of symptoms when eating spicy foods (2/5; 40% vs 4/72; 5.56%; *p* = 0.046).

## 4. Discussion

Currently, most studies of predictive markers of treatment efficacy in CU focus on antihistamines, omalizumab, and cyclosporine. This study is aimed at identifying clinical and blood biomarkers that are promising predictors of response to use of antihistamines, corticosteroids, LTRA, H_2_ blockers, and a low-histamine diet. We also analyzed serum DAO levels in patients with CU to identify its associations with response to a low-histamine diet. We noted that the response to oral agents and a low-histamine diet among those with CU was associated with a number of biomarkers and factors, including age, sex, duration of the disease, concomitant angioedema, a phenotype of dermatographism, the presence of long-lasting wheals, DAO, Hct levels, ESR levels, eosinophil count, IgE, ANA, and a history of exacerbation when a particular food was consumed. Some of these factors may be useful in predicting the efficacy of treatment. Serum DAO levels may be useful when selecting patients with CU who are good candidates for a low-histamine diet.

In clinical practice, the response to sgAH in patients with CU varies, even though it is the first-line treatment recommended in all phenotypes of CU. Consequently, up to 50% of patients with CU need other therapeutic options [5]. Several studies have been published addressing biomarkers of sgAH efficacy in patients with CU. A high urticarial activity score (UAS-7), longer duration of wheals, eosinopenia, basopenia, increased C5a fraction, lower IgE levels, and autologous serum skin test (ASST) positivity were reported to be biomarkers of sgAH-resistant patients compared to sgAH-sensitive patients [6–8]. Increased levels of D-dimer, fibrinogen, ESR, and C-reactive protein were reported to be predictors of nonresponders to a standard dose of sgAH [9]. Duration of disease was also suggested as a predictive marker of severity due to the greater improvement reported in patients who had CU less than one year [10]. In the present study, having symptoms of CU for more than three years was found to be a clinical marker of poor response to corticosteroid. However, a favorable response to sgAH was likely to be seen in this group. In contrast to the result of a previous study [6], an association of low IgE with an insufficient sgAH response was observed in patients with IgE levels below <300 U/mL alone (OR = 0.98, 95% CI 0.97, 1.00; *p* = 0.006). Meanwhile, an association of high IgE levels with an insufficient sgAH response was observed in patients with CU who had an IgE level > 100 U/mL (OR = 1.00, 95% CI 1.00, 1.01; *p* = 0.029), suggesting that there may be a U-shaped relationship between IgE and insufficient sgAH response. In the present study, no association of insufficient sgAH response with elevated ESR levels or eosinopenia was observed.

Corticosteroids, potent immunosuppressants, can be the only rescue for some severe eruptions. However, resistance to corticosteroids can be seen in some patients with CU. In our study, older age seems to be a predictor of less favorable response to corticosteroid in patients with CU. In this respect, corticosteroid should be cautiously used in patients with CU, particularly older adults, due to not only the susceptibility of potential adverse effects but also because of less favorable efficacy. In clinical practice, when a patient with CU has wheals for a long time, elevated ESR, or positive ANA levels, underlying autoimmune diseases usually come to mind, which can be an indication for a corticosteroid. In fact, we found that patients with CU who had an ANA titer ≥ 1 : 80 were more likely to have a long duration of wheals (4/12; 33.33% vs 5/66; 7.58%; *p* = 0.010). However, the association of resistance to corticosteroid with ANA titers ≥ 1 : 80 was also observed. In the meantime, we have not observed the association of responders to corticosteroid with a long duration of wheals or elevated ESR. A possible explanation could be that ANA may be another marker of CU severity that results in resistance to corticosteroids at a lower dose prescribed. Unlike allergic diseases, an increased eosinophil count may not be helpful in selecting patients with CU who would be good candidates for corticosteroids.

Even though LTRA has been recommended as a second-line treatment of CU in the BSACI guideline [1], its efficacy was found to be less reliable in a survey among clinicians in UK. According to our previous survey, 63.2% of allergists/immunologists and 71.7% dermatologists stated that less than 25% of patients with CU were able to benefit from LTRA [11]. However, in real-world experience, there is a subgroup of patients with CU who can benefit from montelukast. Akoglu et al. have reported a significant decreased urinary LTE4 after a low-pseudoallergen diet in responders [12]. In our study, we observed an association of LTRA efficacy with response to a low-histamine diet, suggesting a link between histamine and leukotriene.

Dietary intervention has been reported to be beneficial in patients with CU. The response rate of an elimination diet among patients with CU varied widely in different studies, ranging from 31% to 100% [13]. A recent study focusing on a low-histamine diet for CU reported a 75% response rate [14].

Adherence is often a major issue in dietary intervention. A biomarker for the prediction of efficacy would be required to prompt patients with CU to adhere to the dietary recommendations. In comparison with healthy controls, significantly lower DAO levels in individuals with histamine intolerance benefiting from a histamine-limited diet have been reported (mean 7.04 and SD 6.90 U/mL vs mean 39.50 and SD 18.16 U/mL; *p* = 0.003). However, patients with CU were excluded from the study [15]. In our study, no differences in response to dietary intervention could be observed between groups when the manufacturer's recommended cutoff was used. However, when we used the median of DAO levels as the cutoff, the difference in response to dietary intervention reached statistical significance between groups. Therefore, DAO appears to be a potential biomarker for the efficacy of a low-histamine diet in patients with CU. However, the cutoff level in clinical practice has yet to be determined. Interestingly, an association of DAO insufficiency with favorable response to sgAH and H_2_ blockers was also observed in patients with CU, suggesting antagonists of H_1_ and H_2_ receptors might be helpful in dealing with excessive histamine when histamine metabolism is impaired. However, their efficacy may be insufficient when the capacity of histamine catabolism is excessively impaired, because the association of insufficient sgAH response with low DAO was observed in patients with DAO levels < 10 U/mL (OR = 0.74, 95% CI 0.56, 0.97; *p* = 0.031).

Hemoglobin and Hct level analysis were included in this study because polycythemia could result in increased histamine formation. In the present study, an association of low Hct level with histamine tolerance was observed. Based on the inverse correlation between DAO and Hb, DAO consumption by excessive histamine in the process of erythrocyte metabolism might be considered. Additionally, a positive correlation was also observed between ESR, an acute phase reactant affected by erythrocyte number, and the level of DAO. Daschner et al. reported a significant correlation between IL-6, a proinflammatory cytokine, and DAO levels in patients with CU who were Anisakis sensitization (Rho = 0.57; *p* = 0.003) [15], which could be an alternative explanation for the significant correlation between DAO and ESR. While IL-6 and C-reactive protein have been reported to be biomarkers of CU severity, [7] DAO was reported as not associated with UAS [16].

Theoretically, pseudoallergic reactions are not evident immediately after consuming histamine-rich foods because the symptoms principally result from a buildup of histamine. However, exacerbation of CU followed by specific food intake has been reported by a number of patients. In the study of Magerl et al., fewer than 10% of patients with chronic spontaneous urticaria had reported food as a trigger [17]. In our study, 55.1% of patients with CU reported exacerbations of symptoms when they consumed specific foods. In our real-world experience, it could be difficult to identify the association between food consumption and CU exacerbation in the absence of adequate treatment, including sgAH and dietary intervention. In theory, any food rich in histamine may trigger the eruption in patients with histamine intolerance. As a result, a history of eruption with multiple food triggers might be another predictor of low-histamine diet efficacy. Food that can be a trigger for a CU attack might be a source of pseudoallergens, including salicylate, histamine, and other ingredients not clearly defined. Of course, IgE-mediated food allergy combined with CU should be ruled out. The food trigger reported the most frequently in our study was shrimp. Shrimp is a commonly seen as food allergen, while shellfish has been reported as a histamine releaser, as well as being a source of histamine [18]. Pseudoallergic reaction was preferred to food allergy, based on the association of low-histamine diet efficacy with CU exacerbated by shrimp consumption. Interestingly, reported foods that trigger exacerbation of CU were not on the top of the histamine-rich food list. Notably, most of these foods had been classified as histamine releasers [19]. Compared to histamine-rich foods, histamine-releasing foods may be more likely to immediately trigger pseudoallergic reactions.

Instead of chronic spontaneous urticaria (CSU), dermatographic urticaria was classified as chronic inducible urticaria (CindU). While there is no identified trigger in patients of CSU, CindU can be triggered by physical stimuli. The differences observed in the efficacy of diet intervention between CSU and dermatographic urticaria suggested that a low-histamine diet may be less likely to be beneficial in CindU compared to CSU. In addition, we observed a trend for a lower prevalence of angioedema in patients with dermatographic urticaria (0/13; 0% vs 14/65; 21.54%; *p* = 0.065) and vice versa. While the association of concomitant angioedema with benefit from a low-histamine diet was observed, patients with dermatographism were less likely to benefit from a low-histamine diet.

## 5. Conclusion

A number of biomarkers identified in our study can be helpful to achieve better CU control. DAO appears to be a novel biomarker in CU, predicting the efficacy of sgAH, H_2_ blocker, and a low-histamine diet. This is not surprising because DAO is the key enzyme in histamine metabolism. Furthermore, with appropriate patient selection, the efficacy of LTRA, H_2_ blockers, and a low-histamine diet in patients with CU should be evaluated with additional studies.

## Figures and Tables

**Table 1:** Characteristic of patients with chronic urticaria (*N* = 78).

Variable	*N* (%)
Age (years), mean (SD)	45.8 (13.5)
Sex	
Female	54 (69.2)
Male	24 (30.8)
Duration of disease (years)	
≤ 3	61 (78.2)
>3	17 (21.8)
Diamine oxidase (U/mL), median (IQR)	5.40 (5.23)
Response to second-generation antihistamines	
Good response	44 (57.9)
Partial response	30 (39.5)
No response	2 (2.6)
Response to corticosteroid	
Good response	14 (70.0)
Poor response	6 (30.0)
Response to montelukast	
Good response	6 (54.5)
Poor response	5 (45.5)
Response to H_2_ blocker	
Good response	3 (37.5)
Poor response	5 (62.5)
Response to low-histamine diet	
Good response	55 (76.4)
Poor response	17 (23.6)
Contaminant angioedema	
Presence	13 (16.7)
Absence	65 (83.3)
Predominant manifestation	
Dermatographism	14 (17.9)
Chronic spontaneous urticaria	64 (82.1)
Long-lasting wheals	
Presence	9 (11.5)
Absence	69 (88.5)
Erythrocyte sedimentation rate (mm/hour), median (IQR)	7.00 (10.25)
Eosinophil count (/*μ*L), median (IQR)	130.0 (96.5)
IgE (IU/mL), median (IQR)	103.0 (254.0)
Antinuclear antibody	
≤ 1 : 40	66 (84.6)
≥ 1 : 80	12 (15.4)
Hemoglobin (g/dL), mean (SD) (*N* = 77)	13.61 (1.61)
Hematocrit (%), mean (SD) (*N* = 75)	41.0 (4.1)
Specific food trigger	
Presence	43 (55.1)
Absence	35 (44.9)
Exacerbation when NSAID use	
Presence	4 (5.1)
Absence	74 (94.9)

IQR: interquartile range; NSAID: nonsteroidal anti-inflammatory drug; SD: standard deviation.

**Table 2:** Univariate analyses of mean diamine oxidase level in patients with chronic urticaria.

Variable	Mean (standard deviation)	*p*
Sex		0.004
Male	5.01 (2.85)	
Female	10.68 (13.30)	
Age (years)		0.033
>50	5.93 (5.24)	
≤ 50	10.71 (13.63)	
Predominant manifestation		0.006
Dematographic urticaria	4.98 (2.48)	
Chronic spontaneous urticaria	9.78 (12.42)	

**Table 3:** Age- and sex-adjusted logistic regression analyses for the identification of predictor of treatment efficacy in patients with chronic urticaria.

Variable	Good response to sgAH	Corticosteroid responder	LTRA responder	H_2_ blocker responder	Low-histamine diet responder
	aOR (95% CI)	aOR (95% CI)	aOR (95% CI)	aOR (95% CI)	aOR (95% CI)
Sex					
Female	1	1	1	1	1
Male	0.80 (0.30–2.13)	5.28 (0.39–756.73)	11.59 (0.39–347.44)	1.74 (0.05–57.74)	1.58 (0.45–5.50)
Age (years)	0.99 (0.96–1.02)	0.94 (0.85–1.02)	1.03 (0.92–1.15)	1.15 (0.86–1.53)	1.01 (0.97–1.05)
Age > 50 years	1.53 (0.59–3.99)	**0.02 (0.01–0.92)**	1.16 (0.07–20.28)	7.00 (0.23–1316.85)	1.58 (0.49–5.12)
Duration of chronic urticaria > 3 years	**4.39 (1.14–16.99)**	**0.06 (0.00–0.96)**	0.07 (0.00–1.99)	NC	0.33 (0.10–1.08)
Concomitant angioedema	1.84 (0.51–6.68)	0.34 (0.01–6.27)	3.47 (0.11–668.48)	0.05 (0.00–4.79)	**10.56 (1.28–1374.71)**
Predominant manifestation					
Chronic spontaneous urticaria	1	1	1	1	1
Dermatographism	0.70 (0.21–2.28)	0.56 (0.03–8.87)	0.06 (0.00–1.17)	12.17 (0.72–1972.39)	**0.23 (0.06–0.86)**
Long-lasting wheals	0.32 (0.07–1.43)	0.34 (0.03–2.99)	40.05 (0.52–37458.92)	0.05 (0.00–4.79)	2.26 (0.25–20.42)
Specific food trigger	0.48 (0.18–1.27)	0.08 (0.00–1.27)	0.23 (0.01–10.29)	12.48 (0.67–1736.32)	2.39 (0.76–7.54)
Multiple food triggers	0.54 (0.20–1.48)	0.71 (0.08–5.77)	7.71 (0.61–1011.31)	6.11 (0.04–902.15)	**11.69 (1.42–96.35)**
Low-histamine diet responder	0.47 (0.15–1.53)	2.88 (0.27–45.11)	**67.29 (2.07–64666.59)**	0.18 (0.01–6.32)	NC
Diamine oxidase	0.96 (0.91–1.02)	1.03 (0.96–1.20)	0.50 (0.22–1.14)	0.86 (0.00–1.17)	0.97 (0.93–1.02)
<10 vs ≥ 10 U/mL	**3.90 (1.07–14.25)**	0.53 (0.04–5.13)	NC	2.47 (0.08–443.63)	0.75 (0.17–3.33)
<5.4 vs ≥ 5.4 U/mL	1.09 (0.43–2.76)	1.28 (0.15–13.67)	11.18 (0.30–414.46)	**71.95 (1.55–89411.09)**	**3.78 (1.06–13.46)**
Erythrocyte sedimentation rate	0.99 (0.94–1.05)	1.01 (0.93–1.10)	0.80 (0.51–1.28)	0.73 (0.62–1.39)	1.04 (0.96–1.12)
IgE	1.00 (1.00–1.00)	1.00 (1.00–1.01)	1.00 (1.00–1.01)	0.97 (0.93–1.02)	1.00 (1.00–1.00)
Eosinophil count	1.00 (1.00–1.00)	1.01 (0.99–1.36)	1.00 (0.98–1.01)	0.99 (0.96–1.00)	1.00 (1.00–1.00)
Hematocrit	1.01 (0.87–1.18)	0.38 (0.10–1.45)	0.71 (0.33–1.49)	1.58 (0.94–13.50)	1.14 (0.95–1.38)
< 36% vs ≥ 36%	1.15 (0.28–4.69)	3.93 (0.21–611.55)	NC	0.37 (0.00–11.39)	**0.19 (0.04–0.80)**
ANA ≥ 1 : 80 vs < 1 : 80	1.02 (0.29–3.57)	**0.03 (0.00–0.39)**	7.16 (0.45–16564.22)	NC	1.51 (0.29–7.90)

Adjusted odds ratios with a *p* value < 0.05 are shown in bold. ANA: antinuclear antibody; CI: confidence interval; LTRA: leukotriene receptor antagonist; NC: not calculable; NSAID: nonsteroidal anti-inflammatory drug; OR: odds ratio; sgAH: second-generation antihistamines.

## Data Availability

The data used to support the findings of this study are included within the article.

## Additional Points

*IRB Statement*. The study protocol was approved by the Institutional Review Board of Dalin Tzu Chi Hospital, Buddhist Tzu Chi Medical Foundation (No. B10902014-2) or Taipei Tzu Chi Hospital, Buddhist Tzu Chi Medical Foundation (No. 09-C-095).
